# *In-vivo* biological activity and glycosylation analysis of a biosimilar recombinant human follicle-stimulating hormone product (Bemfola) compared with its reference medicinal product (GONAL-f)

**DOI:** 10.1371/journal.pone.0184139

**Published:** 2017-09-07

**Authors:** Renato Mastrangeli, Abhijeet Satwekar, Francesca Cutillo, Cinzia Ciampolillo, Wolf Palinsky, Salvatore Longobardi

**Affiliations:** 1 Biotech Development Programme, Merck Serono S.p.A. (an affiliate of Merck KGaA, Darmstadt, Germany), Guidonia Montecelio, Rome, Italy; 2 Pharamceutical & Analytical Development Biotech Products, Merck Serono S.p.A. (an affiliate of Merck KGaA, Darmstadt, Germany), Guidonia Montecelio, Rome, Italy; 3 Analytical BQC, Merck RBM S.p.A. (an affiliate of Merck KGaA, Darmstadt, Germany), Ivrea, Turin, Italy; 4 Biotech Development Programme, Merck Biopharma (an affiliate of Merck KGaA, Darmstadt, Germany), Aubonne, Switzerland; 5 Global Medical Affairs Fertility, Merck Serono S.p.A. (an affiliate of Merck KGaA, Darmstadt, Germany), Guidonia Montecelio, Rome, Italy; Swiss Institute of Bioinformatics, SWITZERLAND

## Abstract

Recombinant human follicle-stimulating hormone (r-hFSH) is widely used in fertility treatment. Although biosimilar versions of r-hFSH (follitropin alfa) are currently on the market, given their structural complexity and manufacturing process, it is important to thoroughly evaluate them in comparison with the reference product. This evaluation should focus on how they differ (e.g., active component molecular characteristics, impurities and potency), as this could be associated with clinical outcome. This study compared the site-specific glycosylation profile and batch-to-batch variability of the *in-vivo* bioactivity of Bemfola, a biosimilar follitropin alfa, with its reference medicinal product GONAL-f. The focus of this analysis was the site-specific glycosylation at asparagine (Asn) 52 of the α-subunit of FSH, owing to the pivotal role of Asn52 glycosylation in FSH receptor (FSHR) activation/signalling. Overall, Bemfola had bulkier glycan structures and greater sialylation than GONAL-f. The nominal specific activity for both Bemfola and GONAL-f is 13,636 IU/mg. Taking into account both the determined potency and the nominal amount the average specific activity of Bemfola was 14,522 IU/mg (105.6% of the nominal value), which was greater than the average specific activity observed for GONAL-f (13,159 IU/mg; 97.3% of the nominal value; p = 0.0048), although this was within the range stated in the product label. A higher batch-to-batch variability was also observed for Bemfola versus GONAL-f (coefficient of variation: 8.3% vs 5.8%). A different glycan profile was observed at Asn52 in Bemfola compared with GONAL-f (a lower proportion of bi-antennary structures [~53% vs ~77%], and a higher proportion of tri-antennary [~41% vs ~23%] and tetra-antennary structures [~5% vs <1%]). These differences in the Asn52 glycan profile might potentially lead to differences in FSHR activation. This, together with the greater bioactivity and higher batch-to-batch variability of Bemfola, could partly explain the reported differences in clinical outcomes. The clinical relevance of the differences observed between GONAL-f and Bemfola should be further investigated.

## Introduction

Follicle-stimulating hormone (FSH) is a glycoprotein hormone, synthesized in the hypophysis, that plays a key role in the proliferation and development of ovarian granulosa cells in women and Sertoli cells in men[1, 2]. Exogenous FSH is therefore used clinically to induce multiple follicular development in women, as part of assisted reproductive technologies (ART) such as ovulation induction and controlled ovarian stimulation, and to treat infertility due to gonadotropin deficiency in men [2].

FSH consists of two subunits, a 92-amino acid α subunit and a 111-amino acid β subunit [3]. The α subunit is identical in FSH, luteinizing hormone, human chorionic gonadotropin and thyroid-stimulating hormone, and has been proposed to be of evolutionary significance as there is a high degree of structural conservation among different mammalian species [4]. In contrast, the β subunit is hormone specific [3].

The use of recombinant technology to manufacture FSH in the early 1990s enabled the production of highly purified preparations of recombinant-human FSH (r-hFSH; follitropin alfa and follitropin β) with high batch-to-batch consistency in FSH content, isoform profile and specific activity. Furthermore, follitropin alfa (GONAL-f; Merck KGaA, Darmstadt, Germany) can be filled by mass (FbM), providing very low batch-to-batch variability (< 2%) [5] and enabling more precise dosing [6]. This reduced variability might improve both convenience and effectiveness during stimulation cycles [6]. In 2013, the first biosimilar follitropin alfa was authorized in the European Union (Ovaleap; Teva Pharma B.V., Utrecht, The Netherlands) [7], with a second biosimilar follitropin alfa authorized in 2014 (Bemfola; Finox Biotec AG, Balzers, Liechtenstein) [8].

Differences in glycosylation have an effect on *in-vivo* potency and the biological activity of FSH molecules. The α chain of FSH is glycosylated at asparagine 52 (Asn52) and Asn78, while the β subunit can be glycosylated at Asn7 and Asn24, with the glycosylation profile of each subunit playing a critical role in the activity and clearance of FSH [9]. Glycosylation of the α chain at Asn52 plays an important role in assembly of the functional FSH heterodimer and its subsequent stability [10], and has also been shown to play a pivotal role in FSHR activation/signalling [9, 11–14]. Glycosylation at this site is therefore considered to be essential for bioactivity. In addition, the glycosylation pattern on both subunits contributes to the net *in-vivo* potency of the molecule, with overall glycosylation affecting metabolic clearance [4, 10, 12, 13, 15].

Endogenous pituitary-derived human FSH (hFSH) contains a heterogeneous population of glycovariants, with varying β-subunit site occupancy (the presence or absence of oligosaccharides at a specific site) and glycan antennarity (the branching of the oligosaccharides) [15–17]. The degree of terminal sialylation, sulfation, antennarity and core fucosylation differs in both the α and β subunits. All of these variations can affect the *in-vivo* biological activity of the hormone [15–17]. There are four naturally occurring glycoforms of hFSH, which differ in their glycosylation content (occupancy) and the antennarity of the β subunit[18–23]. These are tetra-glycosylated hFSH24, tri-glycosylated hFSH21 and hFSH18, and di-glycosylated hFSH15. The different glycoforms can be identified according to their migration properties observed by SDS-PAGE Western blotting. The difference in migration properties between hFSH21 and hFSH18 reflects a greater number of antennary structures at Asn7 compared with Asn24 on the β subunit [9, 24]. In addition, tetra-antennary glycan structures have only been reported at Asn7 [9, 24].

The glycoform composition of hFSH in the circulation fluctuates in a characteristic manner throughout the follicular phase. During the early follicular phase, the more acidic hFSH glycoforms (with a higher sialic acid content) prevail, with a shift to less-acidic glycoforms mid-cycle (i.e. during the peri-ovulatory phase) [25–27]. Furthermore, di-glycosylated hFSH is more abundant in younger women, whereas tetra-glycosylated and highly sialylated forms are more predominant in peri/postmenopausal women [16, 19, 21–23, 27]. This suggests that the glycoform composition of circulating hFSH is functionally relevant in cycling women, reflecting their endocrine status.

The FSHR is present as a functional homotrimer [11], and interaction of FSH with the FSHR causes the activation of a complex signalling network, including, but not limited to, the Gs/cAMP pathway, with the Asn52 glycoform composition of hFSH potentially affecting this signalling [28]. This activation affects ovarian function by initiating and mediating a multitude of effects required for reproductive processes, and therefore the developmental competency of the oocyte [29]. This developmental competency also has an impact on pre-implantation, implantation and clinical pregnancy rates [30]. For example, 80% of mouse follicles exposed to the less-acidic human FSH isoforms for 3 days developed into two-cell embryos after *in-vitro* maturation/*in-vitro* fertilization, whereas only 60% of follicles exposed to the acidic isoform for 5 days developed into two-cell embryos [31]. A meta-analysis evaluating two pure FSH preparations (Metrodin-HP [highly purified urinary FSH]; Merck KGaA, Darmstadt, Germany; and GONAL-f), which differed in their glycoform profiles (Metrodin-HP, highly acidic; GONAL-f; less acidic), found that patients treated with GONAL-f performed better than those treated with Metrodin-HP, especially in terms of the number of follicles, the number of oocytes retrieved, and the duration of gonadotropin treatment [32]. These studies highlight the relevance of the glycosylation profile of FSH to its biological functions.

In this study, we report the comparison of the site specific glycosylation profile and the *in-vivo* biological activity of biosimilar follitropin alfa (Bemfola) and its reference medicinal product (GONAL-f), with the aim of comparing relevant attributes that may affect overall FSH bioactivity.

## Materials and methods

### Materials

Urea, iodoacetamide (IAM) and dithiothreitol (DTT) were purchased from Sigma (St. Louis, MO, USA). Chymotrypsin (sequencing grade) was purchased from Roche Diagnostics (Mannheim, Germany). Tris-(hydroxymethyl)-aminomethane (TRIS), ethylenediaminetetraacetic acid (EDTA), trifluoroacetic acid (TFA), hydrochloric acid (HCl) and liquid chromatography–mass spectrometry (LC-MS)-grade acetonitrile (ACN) were obtained from Merck (Darmstadt, Germany). Guanidine 8 M solution was purchased from Thermo Fisher Scientific (San Jose, CA, USA). All water used in experiments was purified with a Milli-Q system from Merck Millipore (Milford, MA, USA). Amicon Ultracel 3k centrifugal filters were purchased from Merck Millipore (Tullagreen, Carrigtwohill, Ireland) and the Acquity UPLC BEH glycan column 1.7μm, 2.1 x 150 mm was purchased from Waters (Milford, MA, USA).

GONAL-f was provided by Merck KGaA (Darmstadt, Germany) and Bemfola batches were purchased in three different markets (Poland, Germany and the United Kingdom). Three batches of GONAL-f (199F005, 199F049, 199F051) and two batches of Bemfola (PPS30403, PNS30226) were used for glycopeptides mapping (**Table 1**), and eight batches of Bemfola were tested for bioactivity (PNS30388, PNS30230, PNS30400, PNS30021, PNS30390, PNS30228, PNS30389 and PNS30229B; **Table 1**).

**Table 1:** Bemfola and GONAL-f batches tested.

Product	Batch number	Nominal IU	Nominal μg	Nominal specific activity IU/mg	Expiry date	Months to expiry	Assay
Bemfola	PNS30388	300	22	13,636	January 2017	13	*+In-vivo* potency
Bemfola	PNS30230	450	33	13,636	September2016	9	
Bemfola	PPS30400	150	11	13,636	September2017	21	
Bemfola	PPS30021	75	5.5	13,636	January 2017	13	
Bemfola	PNS30390	150	11	13,636	February 2017	14	
Bemfola	PNS30228	225	16.5	13,636	September 2016	9+	
Bemfola	PNS30389	225	16.5	13,636	January 2017	13	
Bemfola	PNS30229B	300	22	13,636	September 2017	9	
Bemfola	PNS30226	75	5.5	13,636	September 2017	9	Glycopeptide mapping*
Bemfola	PPS30403	450	33	13,636	September 2017	21	
GONAL-f	199F005	300	22	13,636	July 2017	19	Glycopeptide mapping
GONAL-f	199F049	75	5.5	13,636	August 2017	20	
GONAL-f	199F051	75	5.5	13,636	September 2017	21	

*The batches were available for glycopeptide analysis after the *in-vivo* potency analyses were done. The batches used for glycopeptide analysis were not retested for *in-vivo* potency owing to ethical considerations (to reduce the number of animals used in the testing) and owing to the proven batch-to-batch consistency of the marketed product.

### Glycopeptide mapping

#### Reduction, alkylation and enzymatic digestion

Individual batches of GONAL-f and Bemfola were concentrated to approximately 1 mg/mL using Amicon Ultracel 3k centrifugal filters. Following concentration, 200 μL of protein was then suspended in 200 μL of denaturation buffer containing 8 M gua nidine-HCL, 130 mM Tris-HCl and 1 mM EDTA at pH 7.6, and reduction was performed by adding 20 μL of 500 mM DTT and stirring for 30 minutes at 37°C. The samples were subsequently alkylated by adding 25 μL of 500 mM IAM and stirring in the dark for 30 minutes at room temperature. The buffer containing the reduced and alkylated samples was then exchanged for a digestion buffer containing 2 M urea and 50 mM Tris-HCl at pH 8.0 by using Amicon Ultracel 3k centrifugal filters. The proteins in the samples were then digested at 37°C for 4 hours using chymotrypsin, with an enzyme:substrate ratio of 1:20.

#### Hydrophilic interaction chromatography and mass spectrometry analysis

Chymotrypsin digested protein was analysed using a Synapt G1 mass spectrometer (Waters, Milford, MA,USA) equipped with an Acquity UPLC system (Waters, Milford, MA, USA). The peptides were separated on an Acquity glycan BEH amide UPLC column (1.7μm, 2.1x 150 mm), owing to the high resolving separation for glycosylated peptides and the enhanced peptide retention and separation of peptides from glycopeptides attained with this method[33], and eluted with a mixture of 0.1% TFA in water and 0.1% TFA in ACN. A 30–55% gradient of 0.1% TFA in water over 60 minutes with 0.2 mL/min flow rate was used to separate the glycopeptides. Separated site-specific glycopetide populations (different peptides, various attached glycans) as well as the different site-specific glycopeptides population (same peptide, various attached glycans) were identified and quantified by mass spectrometry.

Mass spectrometry was performed using the MS^E^ function for dataset acquisition in the data independent mode. MS^E^ function uses an intelligent approach and acquires alternating scans with low and high collision energies to obtain precursor ion information, as well as collision-induced dissociation (CID) fragmentation data. The instrument was operated with the following parameters: capillary voltage 3 kV, sampling cone 28 V, extraction cone 4 V, source temperature 100°C, desolvation temperature 350°C, cone gas flow 50 L/H, desolvation gas flow 800 L/H, and scan range 100–2000 m/z.

The mass spectrometry data were analysed by MassLynx 4.1 software (Waters, Milliford, MA, USA). The identity of the N-glycopeptides was manually assigned (S1 Table), and the m/z specific ion counts were determined by the extraction ion chromatogram (XIC). Identified glycopeptides were grouped as per the *N*-glycan site, and the relative distribution of the site-specific *N*-glycan species was calculated based on ion counts. This strategy allows comparison of the relative distribution of the *N*-glycan species attached on the site-specific glycopeptide. Moreover, the use of the unique glycopeptide for the corresponding *N*-glycan site does not significantly create an ionization bias. As the ionization is mostly due to the contribution of the peptide portion, this is the same for each site-specific glycopeptide population (same peptide, different glycans). LC-MS methods with chymotrypsin digest have been reported that detect and quantitate glycan variants [34]. The resulting data from the glycopeptide analysis was further used to calculate the hypothetical charge number (Z-number) expressing the sialic acid content and the Antennarity Index (A-index) (S1 Text) as reported by Gervais et al 2003 [35].

### Determination of bioactivity

The bioactivity of individual batches of Bemfola was evaluated using the Steelman–Pohley in-vivo bioassay, as described in the European Pharmacopoeia 8^th^ Edition 2016 (8.8) 01/2015:2285 (Follitropin) [36]. The bioactivity of individual batches of GONAL-f was obtained from consecutive testing sessions of 22 marketed batches performed in 2015 by the same laboratory, and using the same compendial method and Merck House Reference Standard as the comparator in each analytical session. The Steelman–Pohley *in-vivo* bioassay is based on the measurement of ovarian weight gain in immature female rats co-treated with human chorionic gonadotropin (hCG) and FSH. Female Sprague–Dawley rats, aged 21–22 days, with a weight difference between the heaviest and the lightest rat of not more than 10 g, were obtained from Charles River, Italy.

Bioassays were performed as “3x3 parallel line” assays, with the log-dose response lines of the test preparations of Bemfola, GONAL-f and the Merck House Reference Standard (RHS r-hFSH 2008/01 BIO) (each at three different doses: low, 2 IU FSH/rat; medium, 4 IU FSH/rat; high, 8 IU FSH/rat) with a total volume of 3mL per rat being compared. The Merck House Reference Standard was calibrated against the International Standard for r-hFSH (IS 92/642; 138 IU/ampoule) from the National Institute of Biological Standards (NIBSC). The assigned activity was 147.5 IU/ampoule (with 95% of fiducial limits).

Each of the two test preparations and the Merck House Reference Standard, at each of the three doses, was tested on five immature female rats. On the day after the last injection, rats were euthanized by cervical dislocation following CO_2_ anaesthesia. Ovaries from each rat were removed and weighed. All animals were kept in line with requirements of the current reference legislation. Animal use was reduced to the minimum necessary. The *in-vivo* bioassay was performed under an umbrella authorization (no. 02/2014-UT issued on 3 June 2014), which authorizes the use of rats to perform tests in compliance with the European Pharmacopoeia Monograph requirements for FSH products ‘Follitropin 01/2015:2285 European Pharmacopeia 8.8’ [36]. In addition, the local Animal Welfare Body (Organismo Preposto al Benessere degli Animali) had been informed and agreed to this test in compliance with the European Pharmacopoeia. At the time of the study, no study-specific authorizations were required by the Italian authority.

### Statistical analyses

The *in-vivo* bioassay results were calculated as the weighted mean value from the replicate analyses, using dedicated software (PLA version 3.0 [Stegmann Systems, Germany]). For normalization purposes, the proportions of the nominal value defined on the product label are reported. Differences in the mean (95% confidence interval) potency values for Bemfola (n = 8) and GONAL-f (n = 22) were calculated using Fisher’s least significant difference (F-test). Owing to the between-batch imbalance in the overall analysis, we also performed a cross-validation analysis to confirm the robustness of our conclusions. For these analyses, we randomly selected three groups of GONAL-f samples with similar size to the Bemfola group (Group A [n = 7], Group B [n = 7], and Group C [n = 8]) and compared the mean values for each pair of groups and for each group compared with the mean for the Bemfola group. Significance was calculated using analysis of variance (ANOVA). The statistical analyses of differences in the mean potency values were carried out using Statgraphic version XV (StatPoint Technologies, Warrenton, USA).

## Results

### Glycopeptide mapping

The identification of site-specific glycopeptides and the site-specific pattern distribution (%) by LC-MS are reported in **S1 Table and S2 Table**, respectively. **S2 Table** reports the pattern of *N*-glycan distribution for three GONAL-f and two Bemfola batches, respectively.

#### N-glycan distribution at Asn52 (α chain)

**Fig 1:**
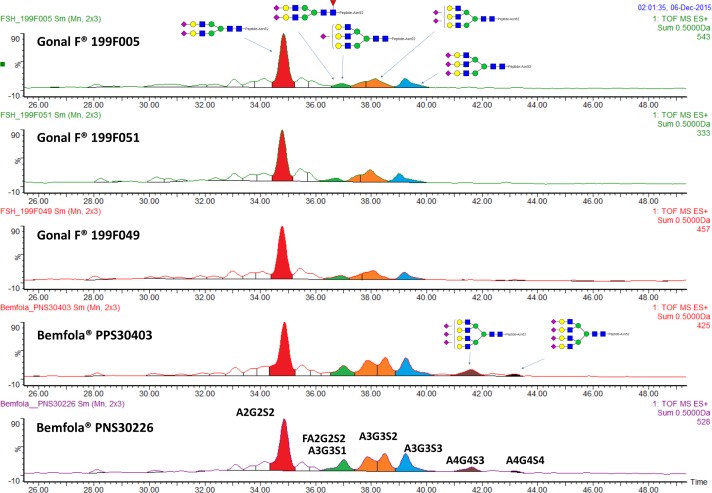
Extracted ion chromatograms of the *N*-glycan distribution at Asn52 for Bemfola and GONAL-f. Fig was constructed using MassLynx 4.1 (Waters, Milford MA, USA) from the respective XIC of the glycopeptides extracted from only Asn52. Asn, asparagine. Blue square, GlcNAc. Green circle, mannose. Yellow circle, galactose. Red triangle. fucose. Purple diamond, sialic acid NeuNAc. Glycan naming: F at the start of the abbreviation indicates a core a(1–6) fucose linked to the inner GlcNAc. Ax indicates the number of antenna (GlcNAc) on trimannosyl core. A2 indicates bi-antennary with both GlcNAcs as b(1–2) linked. A3 indicates tri-antennary with a GlcNAc linked b1-2 to both mannose and a third GlcNAc linked b(1–4) to the a(1–3) linked mannose. A4 indicates tetra-antennary with GlcNAcs linked as A3 with additional GlcNAc b(1–6) linked to a(1–6) mannose. Gx indicates the number (x) of b1-4 linked galactose on the antenna. Sx indicates the number (x) of sialic acids linked to galactose.

**Fig 2:**
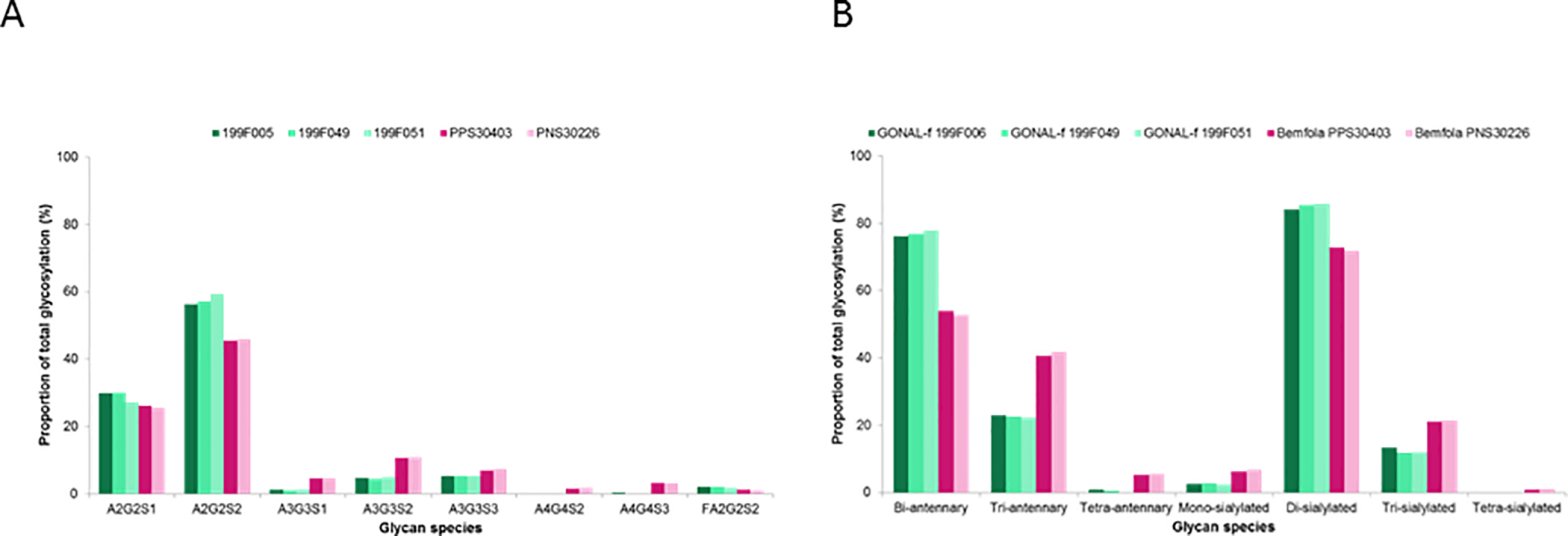
Glycan and antennarity and sialylation distribution at Asn52. **A)** Comparison of glycan distribution at Asn52 between Bemfola and GONAL-f (individual species) **B)** Comparison of antennarity and sialylation at Asn52 between Bemfola and GONAL-f. Asn, asparagine.

The *N*-glycan distribution at Asn52 was consistent across batches of both Bemfola and GONAL-f (**Table 2** and **S2 Table**). However, differences in the distribution of glycans at this site were observed between Bemfola and GONAL-f (**Figs 1** and **2**). The most common glycan species at Asn52 in both products were the less bulky bi-antennary glycans (A2G2S2+A2G2S1+F2A2G2S2); however, they were more abundant in GONAL-f (range 76.2–77.8%) than in Bemfola (range 52.7–53.9%). The more bulky tri-antennary(A3G3S1+A3G3S2+A3G3S3) and tetra-antennary (A4G4S2+A4G4S3) glycans were more abundant in Bemfola (40.7%–41.8% and 5.3–5.5%, respectively) than GONAL-f (22.2–22.9% and 0.0–0.9%, respectively).

**Table 2:** Antennarity, fucoslylation and sialylation at α chain Asn52.

		199F005 GONAL-f	199F049 GONAL-f	199F051 GONAL-f	PPS30403 Bemfola	PNS30226 Bemfola
Antennarity	Bi-antennary	76.2	76.9	77.8	53.9	52.7
	Tri-antennary	22.9	22.6	22.2	40.7	41.8
	Tetra-antennary	0.9	0.5	0.0	5.3	5.5
Fucosylation	A-fucosylated	97.8	97.8	97.6	98.8	98.7
	Fucosylated	2.2	2.2	2.4	1.2	1.3
Sialylation	Mono-sialylated	2.6	2.7	2.4	6.2	6.8
	Di-sialylated	84.0	85.5	85.7	72.8	71.8
	Tri-sialylated	13.4	11.8	11.9	21.0	21.4
	Tetra-sialylated	0.0	0.0	0.0	0.8	1.0

Differences in the distribution of mono-, di-, tri- and tetra-sialylated species were observed between the two products (**Fig 2B**). The ratio of tri- and tetra- versus bi-antennary species was higher in Bemfola samples. However, the higher-antennary species were incompletely sialylated (i.e., one or two out of three, or three out of four antennae bore a sialic acid residue). The hypothetical charge number (Z-number) and antennarity index (A-index) [35]were higher in Bemfola than in GONAL-f (**Table 3**). However, a higher level of incompletely sialylated antennae (e.g., monosialo-tri-antennary glycans) was also observed in Bemfola compared with GONAL-f (green area in **Fig 1**).

**Table 3:** Comparative site-specific and simulated glycan release glycosylation parameters (Z-number and A-index) for GONAL-f and Bemfola.

		199F005 GONAL-f	199F049 GONAL-f	199F051 GONAL-f	PPS30403 Bemfola	PNS30226 Bemfola
α-Asn52	Z-number	211	209	210	216	217
	A-index	225	224	222	251	253
α-Asn78	Z -number	175	174	177	180	180
	A-index	212	211	211	232	232
β- Asn7	Z-number	201	198	200	246	249
	A-index	367	368	367	418	423
β- Asn24	Z-number	172	172	174	198	197
	A-index	209	208	210	240	238
Glycan release simulation	Z-number	190	188	190	210	211
	A-index	253	253	253	285	287

Z-number, hypothetical charge number. A-index, hypotethical antennarity index.

At Asn52, nearly all glycans were present as a-fucosylated complexes in both Bemfola and GONAL-f.

#### N-glycan distribution at Asn78 (α chain)

The most abundant *N-*glycan species at Asn78 was A2G2S2 in both Bemfola and GONAL-f, representing 45.5–45.9% and 56.3–59.4% of the total glycosylation at this site, respectively (**S2 Table**). The second most abundant *N*-glycan species was A2G2S1, representing 25.4–26.1% and 27.2–30.0% of the total glycosylation of Bemfola and GONAL-f, respectively. Overall, the proportion of bi-antennary glycans observed in Bemfola and GONAL-f was 72.3–72.9% and 88.3–89.2%%, respectively. The more bulky tri- and tetra-antennary glycans were more abundant in Bemfola (22.2–22.8% and 4.8–4.9%, respectively) than in GONAL-f (10.8–11.6%% and 0–0.4%, respectively) (**S2 Table, S3 Table, S1 and S2 Figs**).

At Asn78, nearly all glycans were present as a-fucosylated complexes in both Bemfola and GONAL-f.

#### N-glycan distribution at Asn7 (β chain)

N-glycosylation at Asn7 showed the highest heterogeneity, and Asn7 contained glycans that were more bulky and complex than those at the other glycosylation sites. Tetra-antennary glycans and tetra-antennary containing lac-repeat glycans were most abundant at this site, with a higher proportion observed in Bemfola (71.7–74.7%) compared with GONAL-f (47.6–48.4%). The main difference between Bemfola and GONAL-f was the proportion of tetra-antennary glycans containing one lac repeat (Bemfola, 35.7–36.0%; GONAL-f, 15.6–16.6%) and two lac repeats (Bemfola, 5.4–5.8%; GONAL-f, 0.8–1.4%), as well as the proportion of triantennary glycans present (Bemfola, 23.2–24.5%; GONAL-f, 40.4–41.8%). Very low proportions of bi-antennary species were observed in both Bemfola and GONAL-f (0.5–1% and 3.9–4.2%, respectively). The majority of the glycan structures were partially sialylated and fucosylated (**S2 Table, S4 Table, S3 and S4 Figs**).

#### N-glycan distribution at Asn24 (β chain)

Bi-antennary glycans were the most abundant antennary species at this site (Bemfola, 63.8–64.3%, GONAL-f; 70.6–71.4% [**S2 Table, S5 Table, S5 and S6 Figs**). Higher sialylation content was observed for Bemfola compared with GONAL-f, in particular tri-sialylated species (**S2 Table, S5 Table, S6 Fig**). The majority of the glycan species at Asn24 were completely fucosylated complexes.

### Site-specific glycosylation parameters of the α- and β-subunits

The glycosylation parameters (Z-number and A-index) for Bemfola and GONAL-f are reported in **Table 3**. Bemfola has a higher Z-number and a higher antennarity index at all sites, when compared with GONAL-f. In addition to site-specific values, a theoretical Z-number and A-index were calculated for each product as the average of the four site-specific glycan populations, and compared with published data [35]. Greater sialylation, expressed by a higher hypothetical charge number (Z-number), and a higher hypothetical antennarity index (A-index) were observed in the two Bemfola batches (Z-number = 210–211; A-index = 285–287) compared with three GONAL-F batches (Z-number = 188–190; A-index = 253). The reported Z-numbers were in line with European Pharmacopoeia expectation for 2AB detection (Z-number = 177–233) and pulsed amperometric detection (Z-number = 178–274). In addition, the Z-number data calculated for the three batches of GONAL-f in this article are highly consistent with those reported for a r-hFSH drug substance over a period of 3 years (Z-number = 184; A-Index = 255) with variations in the Z-numbers and A-indeces of less than 3.5% and 1%, respectively [35].

### Bioactivity

The average bioactivity observed for Bemfola was within the range stated on the product label (14,403 IU/mg [105.6% of the nominal value]; n = 8, coefficient of variation [CV] 8.3% [S6 Table]). However, most of the observed bioactivity values were higher than stated on the label and were also higher than the average bioactivity values of GONAL-f (13,270 IU/mg [97.3% of the nominal value]; n = 22, CV 5.8% [S6, S7 and S8 Tables]). The p value from the F-test (p = 0.0048 [S9 Table]) was ten-fold less than 0.05, demonstrating a highly statistically significant difference between the average potency of the two products at the 95% confidence level. Furthermore, the statistical power for the two groups (Bemfola, n = 8 and GONAL-f, n = 22) was 75% (S7 Fig) and although the sizes of the two groups were not balanced, the variability within the two groups was similar (Bartlett’s variance check, p value >0.1 [S10 Table]). Since the p value is greater than 0.05, there is not a statistically significant difference between the standard deviations at the 95% confidence level. This allows the comparison of the mean of two groups. A higher batch-to-batch variability was observed for Bemfola (CV 8.3%) compared with GONAL-f (CV 5.8%) (S8 Table). The results from the cross-validation analysis confirmed there were no significant between-group differences among the GONAL-f subgroups (mean proportions of the nominal label value 97.6%, 98.4% and 96.1%, respectively) but the differences between each of the GONAL-f subgroups and the Bemfola group were significant (S11 Table), consistent with the results obtained for the overall analysis (S12 Table). There was homogeneity of variances among the four groups analysed in the cross-validation (Bartlett’s test = 1.19; p value 0.229), meaning there was no statistically significant differences among the standard deviations at the 95% confidence level (S11 Table).

## Discussion

The overall glycosylation profile of FSH plays a role in its clearance, which contributes to the net *in-vivo* potency of the hormone, with the Asn52 glycosylation site of the α-subunit playing a pivotal role in FSHR activation/signalling. Furthermore, FSH is characterized by high glycosylation heterogeneity and complexity, and our results for r-hFSH confirm this.

In our study, significant differences in glycosylation were observed between Bemfola and GONAL-f, with, overall, Bemfola having bulkier glycan structures and a higher sialic acid content compared with GONAL-f. Our results are in agreement with the data reported by Grass *et al* from a Finox study comparing a non-marketed batch of FSH biosimilar with three batches of GONAL-f [14]. The bulkier glycan structures and higher sialylation observed in the study described here suggest that Bemfola should have a longer half-life and, therefore, a higher net *in-vivo* potency compared with GONAL-f.

The FSH glycans at Asn52 have been reported to fit into the central cavity of the homotrimeric FSHR, allowing its separation in monomers, resulting in activation of the FSHR. It has been proposed that smaller and more compact Asn52 glycans can fit into the central cavity of the FSHR homotrimer with greater rapidity compared with bulkier and extended glycans, resulting in a faster response [11]. Bulky tetra-antennary species were detected at Asn52 in Bemfola (5.3–5.5%), whereas only traces (<0.9%) were observed in GONAL-f (**Figs 1** and **2; Table 2**). As pituitary hFSH does not have bulky glycans (e.g. tetra-antennary glycans) at Asn52 [9], these data confirm that at this critical site, GONAL-f more closely resembles pituitary hFSH than Bemfola does.

Although Bemfola has a higher Z-number (which translates to a higher sialylation content) and a higher Antennarity index (which translates to a greater proportion of bulky glycans), than GONAL-f, published data showed similar pharmacokinetic results for these preparations [37]. It is likely that decreasing glomerular filtration caused by the presence of bulkier glycans could be counterbalanced by a higher proportion of non-completed sialylated antennae in Bemfola (see for example the green area in **Fig 1** and **S2 Table** showing the higher level of monosialo-tri-antennary glycans observed in Bemfola compared with GONAL-f), which may accelerate the clearance rate via liver asialoglycoprotein receptors.

In the *in-vivo* potency assay, although the mean activity of Bemfola was within the range stated in the product label, it was significantly higher than GONAL-f (14,403 IU/mL [105.6%] vs 13,159 IU/mL [97.3%]; p = 0.0048). This significant difference in the *in*-*vivo* potency results was replicated in a cross-validation statistical analysis of the potency results for subgroups of the GONAL-f batches comprising seven to eight samples. A higher batch-to-batch variability was also observed for Bemfola compared with GONAL-f (CV = 8.3% vs 5.8%).The biological activity of Bemfola reported here is at the upper end of that reported in its label, and six out of the eight batches tested were higher than the nominal value (13,636 IU/mL [100%]). This may partly explain the stronger and more variable estradiol response observed with Bemfola compared with GONAL-f by Wolzt et al [37]. This may also explain, at least in part, the observed clinical differences between Bemfola and GONAL-f in estradiol production and ovarian hyperstimulation syndrome [38].

Overall the differences observed in glycosylation between Bemfola and GONAL-f, especially at Asn52 of the α chain, might affect FSH pharmacokinetics and FSHR activation and signalling (5–8), and hence be responsible for the difference in bioactivity observed for Bemfola. Further studies are needed to clarify the magnitude of these differences on intracellular signalling and gene expression profile, as well as their clinical relevance, but physicians should be made aware of these differences. According to current ART guidelines, physicians are encouraged to individualize the FSH dose to optimize outcomes. However, as the doses of Bemfola actually administered may differ from those prescribed, owing to the observed variability, this may affect direct and indirect outcomes. Indeed, a Phase III clinical trial comparing Bemfola with GONAL-f found that in the first cycle of treatment, similar numbers of oocytes were recovered, with similar fertilization rates [39]. However, a higher (though not significant) than average estradiol response, an increased rate of ovarian hyperstimulation syndrome, a lower implantation rate, a lower clinical pregnancy rate per embryo transfer, a lower on-going pregnancy rate per embryo transfer and a lower number of patients with live born children were observed with Bemfola compared with GONAL-f. This suggests that there may be clinical differences between Bemfola and GONAL-f. The factors responsible for these differences include the different glycosylation profiles [40] that may potentially affect ovarian response and the subsequent competency of the oocytes.

Because the batches of Bemfola used in the glycopeptide analysis were available only after the in-vivo potency analysis was performed, paired data from the same samples were not available. It was our opinion that, while adding the results from additional analyses would have certainly corroborated and strengthened the results already presented, our data already convincingly and statistically showed that the proportion of glycosylation at the sites most relevant for FSH receptor activation were consistent among batches and that there were clear and consistent differences between the two preparations. In light of this, we felt that the net gain from performing further analyses using more animals could not be justified ethically. However, we do acknowledge the potential for the absence of these analyses to be a limitation to our findings.

In conclusion, differences in the glycosylation profile at the Asn52 site of the α-chain were observed with Bemfola and GONAL-f. Overall, Bemfola showed higher antennarity, higher sialylation and higher batch-to-batch variability in activity compared with GONAL-f. These elements could partly explain the differences in clinical outcomes between Bemfola and GONAL-f reported in the literature. The clinical relevance of these differences should be investigated further, both *in vitro* and *in vivo*.

## Supporting information

S1 Text Glycopeptide mapping. (DOCX)

S1 Table Identity assignment and XIC of the observed m/z ions in the analysed batches. (DOCX)

S2 Table Site-specific glycan pattern distribution (%) by LC-MS analysis in GONAL-f and Bemfola batches. (DOCX)

S3 Table Glycans distribution (%) on Asn78 according to Antennarity, Fucosylation, and Sialylation in GONAL-f and Bemfola batches. (DOCX)

S4 Table Glycan distribution (%) on Asn7 according to Antennarity, Fucosylation, and Sialylation in GONAL-f and Bemfola batches. (DOCX)

S5 Table Glycans distribution (%) on Asn24 according to Antennarity, Fucosylation and Sialylation in GONAL-f and Bemfola batches. (DOCX)

S6 Table Bemfola bioactivity (in vivo). (DOCX)

S7 Table GONAL-f bioactivity (in vivo). (DOCX)

S8 Table Summary statistics for relative % nominal value (site 1 = Bemfola; site 2 = Gonal-f). (DOCX)

S9 Table ANOVA table for final result (Relative %) by site. (DOCX)

S10 Table Variance check. (DOCX)

S11 Table Multiple range tests for cross-validation (Relative %) and variance check per subgroup. (DOCX)

S12 Table Multiple range tests for final result (Relative %) by site. (DOCX)

S1 Fig Antennarity distribution (%) by LC-MS analysis in GONAL-f and Bemfola batches on Asn78. (DOCX)

S2 Fig Sialylation distribution (%) by LC-MS analysis in GONAL- and Bemfola batches on Asn78. (DOCX)

S3 Fig Antennarity distribution (%) by LC-MS analysis in GONAL-f and Bemfola batches on Asn7. (DOCX)

S4 Fig Sialylation distribution (%) by LC-MS analysis in GONAL-f and Bemfola batches on Asn7. (DOCX)

S5 Fig Antennarity distribution (%) by LC-MS analysis in GONAL-f and Bemfola batches on Asn24. (DOCX)

S6 Fig Sialylation distribution (%) by LC-MS analysis in GONAL-f and Bemfola batches on Asn24. (DOCX)

S7 Fig Statistical power. (DOCX)
