# Supplementary material for: In-vivo biological activity and glycosylation analysis of a biosimilar recombinant human follicle-stimulating hormone product (Bemfola) compared with its reference medicinal product (GONAL-f)
Source: PLoS One. 2017 Sep 7;12(9):e0184139. doi: 10.1371/journal.pone.0184139 (PMC5589168; doi:10.1371/journal.pone.0184139)
Supplement: S1 Text — (DOCX) [file pone.0184139.s001.docx]

# S1 Text. Glycopeptide mapping

This section reports the overall results of site-specific distribution patterns (Table in S2 Table). Specific tables and pictures related to glycosylation sites Asn78 on the α chain, and on Asn7 and Asn24 on the β chain are also reported.

Z-number and A-index, were calculated at each site as described by Gervais et al. for the overall released FSH glycans (Gervais et al. 2003). In our case the site specific Z-number and A-index can better characterize the FSH molecule. The Z-number represents the hypothetical charge number (Z), described by Hermentin et al. to characterize protein glycosylation (Hermentin et al. 1996). The A-index represents the hypothetical antennarity index (A). For each site the Z number is defined as the sum of the products of the relative areas (%) of the N-glycan species, each multiplied by the corresponding charge (x= sialic acid). The antennarity index is defined as the sum of the products of the relative areas (%) of the N-glycan species, each multiplied by the corresponding antennae number (n).

*Site specific Z-number*

The algorithm, based on the data reported in table 2 is the following:

Z site_i_ = Σ (% AnGnSx * x) + Σ (% FAnGnSx * x)

*Site specific A-index*

The algorithm, based on the results reported in table 2, is the following:

A site_i_ = Σ (% AnGnSx *n) + Σ (% FAnGnSx * n )

Where:

AnGnSx is a glycoform with antennarity=n, galactose number=n, and sialic acid number= x; n can range from 2 to 4 (for bi-antennary n=2, tri-antennary n=3, tetra-antennary n=4); x can range from 0 to n, and is dependent on the sialic acid content on the glycoform (for a-sialylated x =0, mono-sialylated x=1, di-sialylated x=2, tri-sialylated x=3 and tetra-sialylated x=4).

The term FAnGnSx considers the fucosylated glycoforms.

Site_i_ refers to a specific glycosylation site. In FSH, this can be at α-Asn52, α-Asn78, β-Asn7, and β-Asn24

The simulation of Z-number and A-index as derived from the glycan release methods, can be calculated considering the average value of Z or A.

Z (simulation of glycan release) = (Z_α52_ + Z_α78_+ Z_β7_+ Z_β24_)/4

A (simulation of glycan release) = (A_α52_ + A_α78_+ A_β7_+ A_β24_)/4
