# Supplementary material for: In-vivo biological activity and glycosylation analysis of a biosimilar recombinant human follicle-stimulating hormone product (Bemfola) compared with its reference medicinal product (GONAL-f)
Source: PLoS One. 2017 Sep 7;12(9):e0184139. doi: 10.1371/journal.pone.0184139 (PMC5589168; doi:10.1371/journal.pone.0184139)
Supplement: S1 Table — (DOCX) [file pone.0184139.s002.docx]

# S1 Table. Identity assignment and XIC of the observed m/z ions in the analysed batches

| **RT (by XIC**) | **Observed M/z** | **charge** | **Molecular Mass** | **Theoretical Mass** | **delta ppm** | **glycan type** | **Start** | **End** | **peptide** | **Chain** | **N-Glycan Site** | **Note** | **Peak Volume**  **Gonal F 199F005** | **Peak Volume**  **Gonal F 199F049** | **Peak Volume**  **Gonal F 199F051** | **Peak Volume**  **Bemfola PPS30403** | **Peak Volume**  **Bemfola PNS30226** |
| --- | --- | --- | --- | --- | --- | --- | --- | --- | --- | --- | --- | --- | --- | --- | --- | --- | --- |
| 22.95 | 1143.81 | 3 | 3428.40 | 3428.36 | 14 | A6G2 | 20 | 27 | (F) CISINTTW(C) | beta | Asn24 |  | 11 | 7 | 5 | 1 | N.I. |
| 22.95 | 1715.17 | 2 | 3428.32 | 3428.36 | -10 | A6G2 | 20 | 27 | (F) CISINTTW(C) | beta | Asn24 |  | 6 | 5 | 4 | 1 | N.I. |
| 24.89 | 1143.81 | 3 | 3428.40 | 3428.36 | 14 | A6G2 | 20 | 27 | (F) CISINTTW(C) | beta | Asn24 |  | 22 | 14 | 10 | 2 | 2 |
| 24.89 | 1715.17 | 2 | 3428.32 | 3428.36 | -10 | A6G2 | 20 | 27 | (F) CISINTTW(C) | beta | Asn24 |  | 13 | 10 | 11 | 1 | 1 |
| 25.64 | 1382.09 | 2 | 2762.16 | 2762.10 | 24 | FA2G2 | 20 | 27 | (F) CISINTTW(C) | beta | Asn24 |  | 11 | 6 | 7 | 5 | 7 |
| 26.42 | 1129.79 | 3 | 3386.35 | 3386.47 | -36 | A3G1 | 6 | 19 | (L) TNITIAIEKEECRF(C) | beta | Asn7 |  | 20 | 13 | 8 | 7 | 9 |
| 26.42 | 1694.17 | 2 | 3386.32 | 3386.47 | -43 | A3G1 | 6 | 19 | (L) TNITIAIEKEECRF(C) | beta | Asn7 |  | 12 | 11 | 8 | 5 | 6 |
| 26.45 | 1345.07 | 2 | 2688.12 | 2688.06 | 23 | FA1G1S1_NANA | 20 | 27 | (F) CISINTTW(C) | beta | Asn24 |  | 1 | 2 | 2 | 1 | 1 |
| 26.73 | 1462.99 | 3 | 4385.95 | 4385.83 | 27 | FA2G2S1_NANA | 1 | 19 | NSCELTNITIAIEKEECRF(C) | beta | Asn7 |  | 5 | 3 | 3 | 3 | 1 |
| 26.94 | 1694.17 | 2 | 3386.32 | 3386.47 | -43 | A3G1 | 6 | 19 | (L) TNITIAIEKEECRF(C) | beta | Asn7 |  | 5 | 5 | 5 | 3 | 3 |
| 26.97 | 1129.79 | 3 | 3386.35 | 3386.47 | -36 | A3G1 | 6 | 19 | (L) TNITIAIEKEECRF(C) | beta | Asn7 |  | 8 | 5 | 2 | 3 | 3 |
| 27.20 | 1119.97 | 4 | 4475.85 | 4475.86 | -3 | A3G4 | 1 | 19 | NSCELTNITIAIEKEECRF(C) | beta | Asn7 |  | 4 | 1 | 5 | 1 | N.D |
| 27.20 | 1492.98 | 3 | 4475.92 | 4475.86 | 13 | A3G4 | 1 | 19 | NSCELTNITIAIEKEECRF(C) | beta | Asn7 |  | 21 | 15 | 14 | 2 | 3 |
| 28.05 | 1018.76 | 3 | 3053.26 | 3053.19 | 22 | FA2G2S1 (NANA) | 20 | 27 | (F) CISINTTW(C) | beta | Asn24 | Antennary Fucose: Confirmed by MS MS | 45 | 31 | 20 | 32 | 46 |
| 28.05 | 1527.64 | 2 | 3053.26 | 3053.19 | 24 | FA2G2S1 (NANA) | 20 | 27 | (F) CISINTTW(C) | beta | Asn24 | Antennary Fucose: Confirmed by MS MS | 101 | 75 | 67 | 68 | 87 |
| 28.63 | 1170.27 | 4 | 4677.05 | 4676.92 | 28 | FA2G2S2(NANA) | 1 | 19 | NSCELTNITIAIEKEECRF(C) | beta | Asn7 |  | 6 | 4 | 3 | 1 | N.D |
| 28.63 | 1560.02 | 3 | 4677.04 | 4676.92 | 25 | FA2G2S2(NANA) | 1 | 19 | NSCELTNITIAIEKEECRF(C) | beta | Asn7 |  | 20 | 18 | 15 | 2 | 3 |
| 29.01 | 1362.56 | 3 | 4084.66 | 4084.74 | -20 | F2A5G1 | 6 | 19 | (L) TNITIAIEKEECRF(C) | beta | Asn7 | Antennary Fucose: Confirmed by MS MS | N.I. | 1 | 4 | 1 | N.I. |
| 29.01 | 1294.49 | 4 | 5173.93 | 5174.13 | -39 | F2A5G4 | 1 | 19 | NSCELTNITIAIEKEECRF(C) | beta | Asn7 |  | N.I. | N.I | N.I. | N.I. | N.D |
| 29.01 | 1725.68 | 3 | 5174.02 | 5173.94 | 15 | F2A5G4 | 1 | 19 | NSCELTNITIAIEKEECRF(C) | beta | Asn7 |  | 3 | 2 | 3 | N.D | N.D |
| 29.18 | 1806.75 | 3 | 5417.23 | 5417.28 | -10 | FA3G3S3 (NANA, 2NANA-Ac) | 1 | 19 | NSCELTNITIAIEKEECRF(C) | beta | Asn7 | MS-MS confirmed for acetylated fragment | 3 | 1 | 1 | N.D | N.D |
| 29.72 | 1174.49 | 4 | 4693.93 | 4693.93 | 0 | F2A2G3S1 (NANA) | 1 | 19 | NSCELTNITIAIEKEECRF(C) | beta | Asn7 |  | N.I. | N.I. | N.I. | N.I. | N.D |
| 29.72 | 1565.64 | 3 | 4693.90 | 4693.93 | -7 | F2A2G3S1 (NANA) | 1 | 19 | NSCELTNITIAIEKEECRF(C) | beta | Asn7 |  | 1 | 1 | N.I. | N.I. | N.I. |
| 29.79 | 1725.69 | 3 | 5174.05 | 5174.13 | -16 | F2A5G4 | 1 | 19 | NSCELTNITIAIEKEECRF(C) | beta | Asn7 |  | 3 | 5 | 2 | N.D | N.D |
| 30.26 | 1792.71 | 3 | 5375.11 | 5375.19 | -16 | F3A4G2S2 (NANA) | 1 | 19 | NSCELTNITIAIEKEECRF(C) | beta | Asn7 |  | 4 | 2 | 3 | 3 | N.D |
| 30.26 | 1345.10 | 2 | 2688.18 | 2688.06 | 46 | FA1G1S1_NANA | 20 | 27 | (F) CISINTTW(C) | beta | Asn24 |  | 54 | 46 | 40 | 40 | 45 |
| 30.26 | 1018.77 | 3 | 3053.28 | 3053.19 | 30 | FA2G2S1 (NANA) | 20 | 27 | (F) CISINTTW(C) | beta | Asn24 | Antennary Fucose: Confirmed by MS MS | 12 | 8 | 7 | 10 | 10 |
| 30.26 | 1527.64 | 2 | 3053.26 | 3053.19 | 24 | FA2G2S1 (NANA) | 20 | 27 | (F) CISINTTW(C) | beta | Asn24 | Antennary Fucose: Confirmed by MS MS | 15 | 13 | 10 | 11 | 12 |
| 30.26 | 1115.81 | 3 | 3344.41 | 3344.28 | 38 | FA2G2S2 (NANA) | 20 | 27 | (F) CISINTTW(C) | beta | Asn24 |  | 278 | 211 | 185 | 244 | 319 |
| 30.26 | 1673.20 | 2 | 3344.38 | 3344.28 | 31 | FA2G2S2 (NANA) | 20 | 27 | (F) CISINTTW(C) | beta | Asn24 |  | 215 | 172 | 157 | 172 | 209 |
| 30.71 | 1211.27 | 4 | 4841.05 | 4840.99 | 12 | A4G5 | 1 | 19 | NSCELTNITIAIEKEECRF(C) | beta | Asn7 |  | 6 | 6 | 7 | 3 | 2 |
| 30.71 | 1614.71 | 3 | 4841.11 | 4840.99 | 24 | A4G5 | 1 | 19 | NSCELTNITIAIEKEECRF(C) | beta | Asn7 |  | 21 | 16 | 13 | 5 | 8 |
| 30.91 | 1362.54 | 3 | 4084.60 | 4084.74 | -35 | F2A5G1 | 6 | 19 | (L) TNITIAIEKEECRF(C) | beta | Asn7 | Antennary Fucose: Confirmed by MS MS | 8 | 7 | 5 | 3 | 2 |
| 30.98 | 1792.71 | 3 | 5375.11 | 5375.19 | -16 | F3A4G2S2 (NANA) | 1 | 19 | NSCELTNITIAIEKEECRF(C) | beta | Asn7 |  | 3 | 1 | 3 | 1 | N.D |
| 31.25 | 1633.04 | 3 | 4896.10 | 4895.99 | 22 | A3G3S2 (NANA) | 1 | 19 | NSCELTNITIAIEKEECRF(C) | beta | Asn7 |  | 17 | 14 | 7 | 2 | 4 |
| 31.28 | 1247.54 | 4 | 4986.13 | 4985.99 | 28 | A3G3S2 (NANA) | 1 | 19 | NSCELTNITIAIEKEECRF(C) | beta | Asn7 |  | 17 | 11 | 10 | 10 | 12 |
| 31.28 | 1663.04 | 3 | 4986.10 | 4985.99 | 21 | A3G3S2 (NANA) | 1 | 19 | NSCELTNITIAIEKEECRF(C) | beta | Asn7 |  | 27 | 19 | 20 | 13 | 18 |
| 31.73 | 1261.52 | 4 | 5042.05 | 5042.05 | 0 | FA3G3S2 (NANA) | 1 | 19 | NSCELTNITIAIEKEECRF(C) | beta | Asn7 |  | 3 | 2 | 5 | 2 | 5 |
| 31.83 | 1681.73 | 3 | 5042.17 | 5042.05 | 23 | FA3G3S2 (NANA) | 1 | 19 | NSCELTNITIAIEKEECRF(C) | beta | Asn7 |  | 25 | 22 | 16 | 13 | 17 |
| 32.00 | 1261.52 | 4 | 5042.05 | 5042.05 | 0 | FA3G3S2 (NANA) | 1 | 19 | NSCELTNITIAIEKEECRF(C) | beta | Asn7 |  | 5 | 3 | N.I. | N.I. | N.I. |
| 32.00 | 1681.73 | 3 | 5042.17 | 5042.05 | 23 | FA3G3S2 (NANA) | 1 | 19 | NSCELTNITIAIEKEECRF(C) | beta | Asn7 |  | 25 | 22 | 16 | 13 | 17 |
| 32.17 | 1284.05 | 4 | 5132.17 | 5132.08 | 17 | A4G5S1 (NANA) | 1 | 19 | NSCELTNITIAIEKEECRF(C) | beta | Asn7 |  | 45 | 35 | 22 | 13 | 21 |
| 32.17 | 1711.72 | 3 | 5132.14 | 5132.08 | 11 | A4G5S1 (NANA) | 1 | 19 | NSCELTNITIAIEKEECRF(C) | beta | Asn7 |  | 56 | 42 | 36 | 17 | 22 |
| 32.20 | 1348.54 | 3 | 4042.60 | 4042.69 | -23 | A4G2S1 (NANA) | 6 | 19 | (L) TNITIAIEKEECRF(C) | beta | Asn7 |  | 4 | 8 | 8 | 10 | 15 |
| 32.20 | 1519.37 | 4 | 6073.45 | 6073.38 | 11 | FA4G4S4 (2NANA, 2 NANA-Ac | 1 | 19 | NSCELTNITIAIEKEECRF(C) | beta | Asn7 | MS-MS confirmed for acetylated fragment | N.I | N.I | N.I | N.D | N.D |
| 32.54 | 1297.80 | 4 | 5187.17 | 5187.09 | 15 | A3G3S3 (NANA) | 1 | 19 | NSCELTNITIAIEKEECRF(C) | beta | Asn7 |  | 16 | 13 | 8 | 13 | 18 |
| 32.54 | 1730.05 | 3 | 5187.13 | 5187.09 | 7 | A3G3S3 (NANA) | 1 | 19 | NSCELTNITIAIEKEECRF(C) | beta | Asn7 |  | 19 | 15 | 11 | 13 | 16 |
| 32.64 | 1746.15 | 2 | 3490.28 | 3490.34 | -16 | F2A2G2S2 (NANA) | 20 | 27 | (F) CISINTTW(C) | beta | Asn24 | Antennary Fucose: Confirmed by MS MS | 15 | 11 | 11 | 10 | 13 |
| 32.68 | 1164.46 | 3 | 3490.36 | 3490.34 | 5 | F2A2G2S2 (NANA) | 20 | 27 | (F) CISINTTW(C) | beta | Asn24 | Antennary Fucose: Confirmed by MS MS | 13 | 9 | 11 | 9 | 13 |
| 33.36 | 1334.33 | 4 | 5333.29 | 5333.15 | 26 | FA3G3S3 (NANA) | 1 | 19 | NSCELTNITIAIEKEECRF(C) | beta | Asn7 |  | 70 | 57 | 44 | 25 | 32 |
| 33.36 | 1778.70 | 3 | 5333.08 | 5333.15 | -14 | FA3G3S3 (NANA) | 1 | 19 | NSCELTNITIAIEKEECRF(C) | beta | Asn7 |  | 42 | 32 | 30 | 16 | 18 |
| 33.46 | 1519.37 | 4 | 6073.45 | 6073.38 | 11 | FA4G4S4 (2NANA, 2 NANA-Ac | 1 | 19 | NSCELTNITIAIEKEECRF(C) | beta | Asn7 | MS-MS confirmed for acetylated fragment | 2 | 1 | 1 | N.D | N.D |
| 33.60 | 1505.13 | 2 | 3008.24 | 3008.16 | 28 | A2G2S1 (NANA) | 75 | 83 | (F)KVENHTACH(C ) | alpha | Asn78 |  | 55 | 50 | 36 | 50 | 54 |
| 33.80 | 1237.51 | 3 | 3709.51 | 3709.42 | 23 | FA3G3S2 (NANA) | 20 | 27 | (F) CISINTTW(C) | beta | Asn24 |  | 18 | 13 | 13 | 26 | 37 |
| 34.14 | 1237.51 | 3 | 3709.51 | 3709.42 | 23 | FA3G3S2 (NANA) | 20 | 27 | (F) CISINTTW(C) | beta | Asn24 |  | 8 | 5 | 6 | 13 | 18 |
| 34.17 | 1003.77 | 3 | 3008.29 | 3008.16 | 42 | A2G2S1 (NANA) | 75 | 83 | (F)KVENHTACH(C ) | alpha | Asn78 |  | 171 | 156 | 110 | 150 | 185 |
| 34.68 | 1057.11 | 3 | 3168.31 | 3168.25 | 18 | FA4G2 | 20 | 27 | (F) CISINTTW(C) | beta | Asn24 |  | 13 | 9 | 8 | 13 | 18 |
| 34.96 | 1375.34 | 4 | 5497.33 | 5497.21 | 22 | A5G6S1 (NANA) | 1 | 19 | NSCELTNITIAIEKEECRF(C) | beta | Asn7 |  | 12 | 13 | 5 | 8 | 13 |
| 34.99 | 1389.11 | 4 | 5552.41 | 5552.22 | 34 | A4G4S3 (NANA) | 1 | 19 | NSCELTNITIAIEKEECRF(C) | beta | Asn7 |  | 7 | 5 | 6 | 6 | 9 |
| 34.99 | 1833.39 | 3 | 5497.15 | 5497.21 | -12 | A5G6S1 (NANA) | 1 | 19 | NSCELTNITIAIEKEECRF(C) | beta | Asn7 |  | 9 | 6 | 3 | 6 | 10 |
| 35.19 | 1785.74 | 2 | 3569.46 | 3569.43 | 10 | A2G2S2 (NANA) | 48 | 59 | (M)LVQKNVTSESTC(C ) | alpha | Asn52 |  | 29 | 23 | 15 | 21 | 26.5 |
| 35.19 | 1190.84 | 3 | 3569.50 | 3569.43 | 19 | A2G2S2 (NANA) | 48 | 59 | (M)LVQKNVTSESTC(C ) | alpha | Asn52 |  | 142 | 116 | 80 | 107 | 132 |
| 35.26 | 1237.51 | 3 | 3709.51 | 3709.42 | 23 | FA3G3S2 (NANA) | 20 | 27 | (F) CISINTTW(C) | beta | Asn24 |  | 7 | 1 | 1 | 9 | 10 |
| 35.30 | 1851.75 | 3 | 5552.23 | 5552.22 | 1 | A4G4S3 (NANA) | 1 | 19 | NSCELTNITIAIEKEECRF(C) | beta | Asn7 |  | 4 | 8 | 7 | 2 | 5 |
| 35.43 | 1411.60 | 4 | 5642.37 | 5642.25 | 21 | FA5G6S1 (NANA) | 1 | 19 | NSCELTNITIAIEKEECRF(C) | beta | Asn7 |  | 8 | 11 | 9 | 20 | 28 |
| 35.47 | 1881.75 | 3 | 5642.23 | 5642.25 | -4 | FA5G6S1 (NANA) | 1 | 19 | NSCELTNITIAIEKEECRF(C) | beta | Asn7 |  | 8 | 6 | 6 | 7 | 11 |
| 35.50 | 1334.56 | 3 | 4000.66 | 4000.52 | 35 | FA3G3S3 (NANA) | 20 | 27 | (F) CISINTTW(C) | beta | Asn24 | Antennary Fucose: Confirmed by MS MS | 86 | 65 | 63 | 121 | 167 |
| 35.53 | 1375.34 | 4 | 5497.33 | 5497.21 | 22 | A5G6S1 (NANA) | 1 | 19 | NSCELTNITIAIEKEECRF(C) | beta | Asn7 |  | 15 | 13 | 10 | 20 | 27 |
| 35.53 | 1833.39 | 3 | 5497.15 | 5497.21 | -12 | A5G6S1 (NANA) | 1 | 19 | NSCELTNITIAIEKEECRF(C) | beta | Asn7 |  | 10 | 8 | 10 | 13 | 18 |
| 35.64 | 1851.75 | 3 | 5552.23 | 5552.22 | 1 | A4G4S3 (NANA) | 1 | 19 | NSCELTNITIAIEKEECRF(C) | beta | Asn7 |  | 5 | n.i | n.i | 8 | 10 |
| 35.87 | 1389.11 | 4 | 5552.41 | 5552.22 | 34 | A4G4S3 (NANA) | 1 | 19 | NSCELTNITIAIEKEECRF(C) | beta | Asn7 |  | 7 | 6 | 7 | 14 | 16 |
| 35.94 | 1425.59 | 4 | 5698.33 | 5698.28 | 9 | FA4G4S3 (NANA) | 1 | 19 | NSCELTNITIAIEKEECRF(C) | beta | Asn7 |  | 10 | 8 | 6 | 8 | 10 |
| 35.94 | 1900.39 | 3 | 5698.15 | 5698.28 | -23 | FA4G4S3 (NANA) | 1 | 19 | NSCELTNITIAIEKEECRF(C) | beta | Asn7 |  | 7 | 4 | 3 | 5 | 6 |
| 36.08 | 1003.77 | 3 | 3008.29 | 3008.16 | 42 | A2G2S1 (NANA) | 75 | 83 | (F)KVENHTACH(C ) | alpha | Asn78 |  | 16 | 11 | 7 | 14 | 14 |
| 36.11 | 1448.11 | 4 | 5788.41 | 5788.31 | 17 | A5G6S2 NANA | 1 | 19 | NSCELTNITIAIEKEECRF(C) | beta | Asn7 |  | 41 | 30 | 24 | 28 | 43 |
| 36.11 | 1930.41 | 3 | 5788.21 | 5788.31 | -18 | A5G6S2 NANA | 1 | 19 | NSCELTNITIAIEKEECRF(C) | beta | Asn7 |  | 14 | 10 | 9 | 13 | 14 |
| 36.11 | 1100.79 | 3 | 3299.35 | 3299.25 | 29 | A2G2S2 (NANA) | 75 | 83 | (F)KVENHTACH(C ) | alpha | Asn78 |  | 386 | 347 | 281 | 314 | 393 |
| 36.11 | 1650.69 | 2 | 3299.36 | 3299.44 | -23 | A2G2S2 (NANA) | 75 | 83 | (F)KVENHTACH(C ) | alpha | Asn78 |  | 70 | 66 | 53 | 59 | 65 |
| 36.42 | 1215.54 | 3 | 3643.60 | 3643.42 | 48 | A3G3S1 (NANA) | 48 | 59 | (M)LVQKNVTSESTC(C ) | alpha | Asn52 |  | 3 | N.I | N.I | 3 | 4 |
| 36.55 | 1461.91 | 4 | 5843.62 | 5843.32 | 52 | A4G4S4 (NANA) | 1 | 19 | NSCELTNITIAIEKEECRF(C) | beta | Asn7 |  | 17 | 13 | 10 | 18 | 35 |
| 36.55 | 1730.34 | 3 | 5188.00 | 5188.11 | -22 | F2A3G3S2 (NANA) | 1 | 19 | NSCELTNITIAIEKEECRF(C) | beta | Asn7 | Antennary Fucose: Confirmed by MS MS | N.I | N.I. | N.I. | N.D | N.D |
| 36.55 | 1900.39 | 3 | 5698.15 | 5698.28 | -23 | FA4G4S3 (NANA) | 1 | 19 | NSCELTNITIAIEKEECRF(C) | beta | Asn7 |  | 8 | 4 | 6 | 8 | 13 |
| 36.55 | 1154.14 | 3 | 3459.40 | 3459.35 | 13 | FA4G2S1 (NANA) | 20 | 27 | (F) CISINTTW(C) | beta | Asn24 |  | 31 | 26 | 25 | 28 | 39 |
| 36.59 | 1425.59 | 4 | 5698.33 | 5698.28 | 9 | FA4G4S3 (NANA) | 1 | 19 | NSCELTNITIAIEKEECRF(C) | beta | Asn7 |  | 16 | 11 | 11 | 21 | 32 |
| 36.70 | 1645.65 | 3 | 4933.93 | 4933.98 | -11 | A3G3S3 (NANA) | 48 | 65 | (M)LVQKNVTSESTCCVAKSY(N) | alpha | Asn52 |  | N.D | N.D | N.D | 3 | 5 |
| 37.00 | 1215.54 | 3 | 3643.60 | 3643.42 | 48 | A3G3S1 (NANA) | 48 | 59 | (M)LVQKNVTSESTC(C ) | alpha | Asn52 |  | 3 | 5 | 1 | 12 | 17 |
| 37.13 | 1149.45 | 3 | 3445.32 | 3445.30 | 7 | FA2G2S2 (NANA) | 75 | 83 | (F)KVENHTACH(C ) | alpha | Asn78 |  | 3 | 3 | 1 | 2 | 11 |
| 37.17 | 1498.39 | 4 | 5989.53 | 5989.37 | 27 | FA4G4S4 (NANA) | 1 | 19 | NSCELTNITIAIEKEECRF(C) | beta | Asn7 |  | 47 | 35 | 31 | 31 | 50 |
| 37.17 | 1997.46 | 3 | 5989.36 | 5989.37 | -2 | FA4G4S4 (NANA) | 1 | 19 | NSCELTNITIAIEKEECRF(C) | beta | Asn7 |  | 13 | 9 | 9 | 13 | 15 |
| 37.37 | 1239.53 | 3 | 3715.57 | 3715.64 | -20 | FA2G2S2 (NANA) | 48 | 59 | (M)LVQKNVTSESTC(C ) | alpha | Asn52 |  | 5 | 4 | 3 | 3 | 4 |
| 37.44 | 1687.69 | 2 | 3373.36 | 3373.29 | 22 | A3G3S1 (NANA) | 75 | 83 | (F)KVENHTACH(C ) | alpha | Asn78 |  | N.I. | N.I. | N.I. | 6 | 7 |
| 37.85 | 1125.46 | 3 | 3373.36 | 3373.29 | 20 | A3G3S1 (NANA) | 75 | 83 | (F)KVENHTACH(C ) | alpha | Asn78 |  | 10 | 8 | 7 | 32 | 39 |
| 38.15 | 1312.57 | 3 | 3934.69 | 3934.48 | 53 | A3G3S2 (NANA) | 48 | 59 | (M)LVQKNVTSESTC(C ) | alpha | Asn52 |  | 13 | 16 | 10 | 21 | 27 |
| 38.22 | 1149.45 | 3 | 3445.32 | 3445.30 | 7 | FA2G2S2 (NANA) | 75 | 83 | (F)KVENHTACH(C ) | alpha | Asn78 |  | 14 | 12 | 9 | 9 | n.I. |
| 38.25 | 1723.67 | 2 | 3445.32 | 3445.30 | 7 | FA2G2S2 (NANA) | 75 | 83 | (F)KVENHTACH(C ) | alpha | Asn78 |  | N.I | N.I | N.I | N.I | N.I |
| 38.36 | 1456.24 | 3 | 4365.70 | 4365.64 | 13 | FA4G4S3 (NANA) | 20 | 27 | (F) CISINTTW(C) | beta | Asn24 |  | 7 | 5 | 5 | 14 | 12 |
| 38.80 | 1312.57 | 3 | 3934.69 | 3934.48 | 53 | A3G3S2 (NANA) | 48 | 59 | (M)LVQKNVTSESTC(C ) | alpha | Asn52 |  | 5 | N.I | 4 | 23 | 29 |
| 38.93 | 1516.93 | 4 | 6063.69 | 6063.41 | 46 | FA5G5S3 | 1 | 19 | NSCELTNITIAIEKEECRF(C) | beta | Asn7 |  | 6 | 2 | 3 | 15 | 28 |
| 39.04 | 1553.15 | 4 | 6208.57 | 6208.45 | 19 | A5G5S4 (NANA) | 1 | 19 | NSCELTNITIAIEKEECRF(C) | beta | Asn7 | HexNAc repeat confirmed by MS-MS | N.D | N.D | N.D | 22 | 14 |
| 39.04 | 1456.24 | 3 | 4365.70 | 4365.64 | 13 | FA4G4S3 (NANA) | 20 | 27 | (F) CISINTTW(C) | beta | Asn24 |  | 8 | 5 | 5 | 34 | 26 |
| 39.04 | 1222.51 | 3 | 3664.51 | 3664.53 | -6 | A3G3S2 (NANA) | 75 | 83 | (F)KVENHTACH(C ) | alpha | Asn78 |  | 19 | 14 | 14 | 62 | 79 |
| 39.07 | 1539.37 | 4 | 6153.45 | 6153.44 | 1 | A6G7S2 (NANA | 1 | 19 | NSCELTNITIAIEKEECRF(C) | beta | Asn7 |  | 11 | 5 | 5 | 31 | 45 |
| 39.50 | 1553.15 | 4 | 6208.57 | 6208.45 | 19 | A5G5S4 (NANA) | 1 | 19 | NSCELTNITIAIEKEECRF(C) | beta | Asn7 | HexNAc repeat confirmed by MS-MS | 2 | 1 | N.I. | 13 | 23 |
| 39.51 | 1516.93 | 4 | 6063.69 | 6063.41 | 46 | FA5G5S3 | 1 | 19 | NSCELTNITIAIEKEECRF(C) | beta | Asn7 |  | 4 | 2 | 2 | 13 | 17 |
| 39.58 | 1222.51 | 3 | 3664.51 | 3664.53 | -6 | A3G3S2 (NANA) | 75 | 83 | (F)KVENHTACH(C ) | alpha | Asn78 |  | 20 | 18 | 14 | 25 | 30 |
| 39.65 | 989.91 | 4 | 3955.61 | 3955.49 | 30 | A3G3S3 (NANA) | 75 | 83 | (F)KVENHTACH(C ) | alpha | Asn78 |  | 7 | 7 | 4 | 11 | 14 |
| 39.78 | 1409.59 | 3 | 4225.75 | 4225.66 | 20 | A3G3S3 (NANA) | 48 | 59 | (M)LVQKNVTSESTC(C ) | alpha | Asn52 |  | 26 | 19 | 15 | 32 | 43 |
| 39.78 | 1057.45 | 4 | 4225.77 | 4225.66 | 26 | A3G3S3 (NANA) | 48 | 59 | (M)LVQKNVTSESTC(C ) | alpha | Asn52 |  | 3 | 2 | N.I | 5 | 4 |
| 39.90 | 1589.69 | 4 | 6354.73 | 6354.51 | 34 | FA5G5S4 (NANA) | 1 | 19 | NSCELTNITIAIEKEECRF(C) | beta | Asn7 | HexNAc repeat confirmed by MS-MS | 15 | 10 | 10 | 42 | 50 |
| 39.99 | 1165.22 | 4 | 4656.85 | 4656.74 | 23 | FA4G4S4 (NANA) | 20 | 27 | (F) CISINTTW(C) | beta | Asn24 | Antennary Fucose: Confirmed by MS MS | 2 | 2 | N.I | 4 | 4 |
| 40.60 | 1319.52 | 3 | 3955.54 | 3955.49 | 12 | A3G3S3 (NANA) | 75 | 83 | (F)KVENHTACH(C ) | alpha | Asn78 |  | 36 | 31 | 26 | 46 | 59 |
| 41.52 | 1344.19 | 3 | 4029.55 | 4029.52 | 7 | A4G4S2 (NANA) | 75 | 83 | (F)KVENHTACH(C ) | alpha | Asn78 |  | N.D | N.D | N.D | 10 | 18 |
| 41.86 | 1344.19 | 3 | 4029.55 | 4029.52 | 7 | A4G4S2 (NANA) | 75 | 83 | (F)KVENHTACH(C ) | alpha | Asn78 |  | N.D | N.D | N.D | 3 | N.I. |
| 42.37 | 1531.28 | 3 | 4590.82 | 4590.79 | 6 | A4G4S3 (NANA) | 48 | 59 | (M)LVQKNVTSESTC(C ) | alpha | Asn52 |  | 2 | 1 | N.I | 11 | 14 |
| 42.71 | 1081.19 | 4 | 4320.73 | 4320.63 | 23 | A4G4S3 (NANA) | 75 | 83 | (F)KVENHTACH(C ) | alpha | Asn78 |  | N.D | N.D | N.D | 10 | 8 |
| 43.05 | 1675.02 | 3 | 5022.04 | 5021.87 | 33 | FA5G5S4 (NANA) | 20 | 27 | (F) CISINTTW(C) | beta | Asn24 |  | 2 | N.I | N.I | 11 | 17 |
| 43.18 | 1628.30 | 3 | 4881.88 | 4881.89 | -3 | A4G4S4 (NANA) | 48 | 59 | (M)LVQKNVTSESTC(C ) | alpha | Asn52 |  | N.D | N.D | N.D | 2 | 3 |
| 43.53 | 1441.23 | 3 | 4320.67 | 4320.62 | 11 | A4G4S3 (NANA) | 75 | 83 | (F)KVENHTACH(C ) | alpha | Asn78 |  | 3 | N.I | N.I | 17 | 22 |

### Legends: Glycan type- as per the oxford nomenclature, NANA- N-acetyl neuraminic acid. N.D – not detected, N.I. not integrable
