# Supplementary material for: In-vivo biological activity and glycosylation analysis of a biosimilar recombinant human follicle-stimulating hormone product (Bemfola) compared with its reference medicinal product (GONAL-f)
Source: PLoS One. 2017 Sep 7;12(9):e0184139. doi: 10.1371/journal.pone.0184139 (PMC5589168; doi:10.1371/journal.pone.0184139)
Supplement: S2 Table — (DOCX) [file pone.0184139.s003.docx]

# S2 Table. Site-specific glycan pattern distribution (%) by LC-MS analysis in GONAL-f and Bemfola batches

|  | **GONAL-f** | | | **Bemfola** | |
| --- | --- | --- | --- | --- | --- |
| **Batch number** | 199F005 | 199F049 | 199F051 | PPS30403 | PNS30226 |
| **αAsn52** | | | | | |
| **A2G2S2** | 74.0 | 74.7 | 75.4 | 52.7 | 51.4 |
| **A3G3S1** | 2.6 | 2.7 | 2.4 | 6.2 | 6.8 |
| **A3G3S2** | 7.8 | 8.6 | 7.9 | 18.1 | 18.2 |
| **A3G3S3** | 12.6 | 11.3 | 11.9 | 16.5 | 16.9 |
| **A4G4S3** | 0.9 | 0.5 | 0.0 | 4.5 | 4.5 |
| **A4G4S4** | 0.0 | 0.0 | 0.0 | 0.8 | 1.0 |
| **FA2G2S2** | 2.2 | 2.2 | 2.4 | 1.2 | 1.3 |
| **αAsn78** | | | | | |
| **A2G2S1** | 29.9 | 30.0 | 27.2 | 26.1 | 25.4 |
| **A2G2S2** | 56.3 | 57.1 | 59.4 | 45.5 | 45.9 |
| **A3G3S1** | 1.2 | 1.1 | 1.2 | 4.6 | 4.6 |
| **A3G3S2** | 4.8 | 4.4 | 5.0 | 10.6 | 10.9 |
| **A3G3S3** | 5.3 | 5.3 | 5.3 | 7.0 | 7.3 |
| **A4G4S2** | 0.0 | 0.0 | 0.0 | 1.6 | 1.8 |
| **A4G4S3** | 0.4 | 0.0 | 0.0 | 3.3 | 3.0 |
| **FA2G2S2** | 2.1 | 2.1 | 1.8 | 1.3 | 1.1 |
| **βAsn7** | | | | | |
| **A3G1** | 5.5 | 5.4 | 4.4 | 3.1 | 2.7 |
| **A3G3S2** | 7.5 | 7.0 | 7.1 | 4.3 | 4.4 |
| **A3G3S3** | 4.3 | 4.5 | 3.7 | 4.5 | 4.4 |
| **A3G4** | 3.1 | 2.6 | 3.7 | 0.5 | 0.4 |
| **A4G2S1** | 0.5 | 1.3 | 1.5 | 1.7 | 1.9 |
| **A4G4S3** | 2.8 | 3.0 | 3.8 | 5.2 | 5.1 |
| **A4G4S4** | 2.1 | 2.1 | 1.9 | 3.1 | 4.5 |
| **A4G5** | 3.3 | 3.5 | 3.8 | 1.4 | 1.3 |
| **A4G5S1** | 12.4 | 12.3 | 11.2 | 5.2 | 5.5 |
| **A5G5S4** | 0.2 | 0.2 | 0.0 | 6.1 | 4.7 |
| **A5G6S1** | 4.4 | 5.1 | 3.5 | 5.9 | 6.4 |
| **A5G6S2** | 6.8 | 6.4 | 6.3 | 7.1 | 7.3 |
| **A6G7S2** | 1.4 | 0.8 | 1.0 | 5.4 | 5.8 |
| **F2A2G3S1** | 0.1 | 0.2 | N.I. | N.I. | N.I. |
| **F2A5G1** | 1.0 | 1.3 | 1.7 | 0.7 | 0.3 |
| **F2A5G4** | 0.7 | 1.1 | 1.0 | N.I. | N.I. |
| **FA2G2S1** | 0.6 | 0.5 | 0.6 | 0.5 | 0.1 |
| **FA2G2S2** | 3.2 | 3.5 | 3.5 | 0.5 | 0.4 |
| **FA3G3S2** | 7.1 | 7.8 | 7.1 | 4.9 | 5.0 |
| **FA3G3S3** | 13.8 | 14.2 | 14.2 | 7.1 | 6.4 |
| **FA3G3S1SAc2** | 0.4 | 0.2 | 0.2 | 0.0 | 0.0 |
| **FA4G4S3** | 5.0 | 4.3 | 5.0 | 7.3 | 7.8 |
| **FA4G4S2SAc2** | 0.2 | 0.2 | 0.2 | 0.0 | 0.0 |
| **FA4G4S4** | 7.4 | 7.0 | 7.7 | 7.7 | 8.3 |
| **FA5G5S3** | 1.2 | 0.6 | 1.0 | 4.9 | 5.8 |
| **FA5G5S4** | 1.8 | 1.6 | 1.9 | 7.3 | 6.4 |
| **FA5G6S1** | 2.0 | 2.7 | 2.9 | 4.7 | 5.0 |
| **βAsn24** | | | | | |
| **A6G2** | 5.2 | 4.8 | 4.5 | 0.6 | 0.3 |
| **F2A2G2S2** | 2.8 | 2.7 | 3.3 | 2.2 | 2.3 |
| **FA1G1S1** | 5.5 | 6.4 | 6.2 | 4.7 | 4.1 |
| **FA2G2** | 1.1 | 0.8 | 1.0 | 0.6 | 0.6 |
| **FA2G2S1** | 17.4 | 16.9 | 15.5 | 13.8 | 13.9 |
| **FA2G2S2** | 49.6 | 51.0 | 50.8 | 47.3 | 47.4 |
| **FA3G3S2** | 3.3 | 2.5 | 3.0 | 5.5 | 5.8 |
| **FA3G3S3** | 8.7 | 8.7 | 9.4 | 13.8 | 15.0 |
| **FA4G2** | 1.3 | 1.2 | 1.2 | 1.5 | 1.6 |
| **FA4G2S1** | 3.1 | 3.5 | 3.7 | 3.2 | 3.5 |
| **FA4G4S3** | 1.5 | 1.3 | 1.5 | 5.5 | 3.4 |
| **FA4G4S4** | 0.2 | 0.3 | N.I. | 0.5 | 0.4 |
| **FA5G5S4** | 0.2 | N.I. | N.I. | 1.3 | 1.5 |

N.I., not integrable. SAc, acetylated N-acetyl neuraminic acid
