# Supplementary material for: In-vivo biological activity and glycosylation analysis of a biosimilar recombinant human follicle-stimulating hormone product (Bemfola) compared with its reference medicinal product (GONAL-f)
Source: PLoS One. 2017 Sep 7;12(9):e0184139. doi: 10.1371/journal.pone.0184139 (PMC5589168; doi:10.1371/journal.pone.0184139)
Supplement: S3 Table — (DOCX) [file pone.0184139.s004.docx]

# S3 Table. Glycan distribution (%) on Asn78 according to antennarity, fucosylation, and sialylation in GONAL-f and Bemfola batches

|  |  | 199F005  GONAL-f | 199F049 GONAL-f | 199F051 GONAL-f | PPS30403  Bemfola | PNS30226 Bemfola |
| --- | --- | --- | --- | --- | --- | --- |
| Antennarity | Bi-antennary | 88.3 | 89.2 | 88.4 | 72.9 | 72.3 |
|  | Tri-antennary | 11.4 | 10.8 | 11.6 | 22.2 | 22.8 |
|  | Tetra-antennary | 0.4 | 0.0 | 0.0 | 4.9 | 4.8 |
| Fucosylation | A-fucosylated | 97.9 | 97.9 | 98.2 | 98.7 | 98.9 |
|  | Fucosylated | 2.1 | 2.1 | 1.8 | 1.3 | 1.1 |
| Sialylation | Mono-sialylated | 31.1 | 31.1 | 28.5 | 30.7 | 30.0 |
|  | Di-sialylated | 63.2 | 63.6 | 66.2 | 59.0 | 59.7 |
|  | Tri-sialylated | 5.7 | 5.3 | 5.3 | 10.2 | 10.3 |
