# Supplementary material for: In-vivo biological activity and glycosylation analysis of a biosimilar recombinant human follicle-stimulating hormone product (Bemfola) compared with its reference medicinal product (GONAL-f)
Source: PLoS One. 2017 Sep 7;12(9):e0184139. doi: 10.1371/journal.pone.0184139 (PMC5589168; doi:10.1371/journal.pone.0184139)
Supplement: S4 Table — (DOCX) [file pone.0184139.s005.docx]

# S4 Table. Glycan distribution (%) on Asn7 according to Antennarity, Fucosylation, and Sialylation in GONAL-f and Bemfola batches

|  |  | 199F005  GONAL-f | 199F049 GONAL-f | 199F051 GONAL-f | PPS30403  Bemfola | PNS30226 Bemfola |
| --- | --- | --- | --- | --- | --- | --- |
| Antennarity | Bi-antennary | 3.9 | 4.2 | 4.0 | 1.0 | 0.5 |
|  | Tri-antennary | 41.7 | 41.8 | 40.4 | 24.5 | 23.2 |
|  | Tetra-antennary | 30.5 | 30.2 | 31.3 | 30.3 | 33.2 |
|  | Tetra-antennary (1HexNac repeat) | 16.5 | 16.6 | 15.6 | 36.0 | 35.7 |
|  | Tetra-antennary (2HexNac repeat) | 1.4 | 0.8 | 1.0 | 5.4 | 5.8 |
| Fucosylation | A-fucosylated | 54.4 | 54.2 | 51.9 | 53.7 | 54.4 |
|  | Mono-fucosylated | 42.9 | 42.7 | 44.2 | 44.9 | 45.3 |
|  | Bi-fucosylated | 1.8 | 2.6 | 2.7 | 0.7 | 0.3 |
| Sialylation | Mono-sialylated | 20.1 | 22.1 | 19.6 | 18.1 | 19.0 |
|  | Di-sialylated | 26.0 | 25.6 | 25.0 | 22.3 | 22.8 |
|  | Tri-sialylated | 27.6 | 26.9 | 27.9 | 29.0 | 29.5 |
|  | Tetra-sialylated | 11.8 | 11.0 | 11.7 | 24.2 | 24.0 |
