# Supplementary material for: In-vivo biological activity and glycosylation analysis of a biosimilar recombinant human follicle-stimulating hormone product (Bemfola) compared with its reference medicinal product (GONAL-f)
Source: PLoS One. 2017 Sep 7;12(9):e0184139. doi: 10.1371/journal.pone.0184139 (PMC5589168; doi:10.1371/journal.pone.0184139)
Supplement: S5 Table — (DOCX) [file pone.0184139.s006.docx]

# S5 Table. Glycan distribution (%) on Asn24 according to Antennarity, Fucosylation and Sialylation in GONAL-f and Bemfola batches

|  |  | 199F005  GONAL-f | 199F049 GONAL-f | 199F051 GONAL-f | PPS30403  Bemfola | PNS30226 Bemfola |
| --- | --- | --- | --- | --- | --- | --- |
| Antennarity | Mono-antennary | 5.5 | 6.4 | 6.2 | 4.7 | 4.1 |
|  | Bi-antennary | 70.9 | 71.4 | 70.6 | 63.8 | 64.3 |
|  | Tri-antennary | 12.0 | 11.2 | 12.3 | 19.2 | 20.8 |
|  | Tetra-antennary | 6.1 | 6.3 | 6.4 | 10.6 | 8.9 |
|  | Tetra-antennary (1HexNac repeat) | 0.2 | 0.0 | 0.0 | 1.3 | 1.5 |
|  | Tetra-antennary (2HexNac repeat) | 5.2 | 4.8 | 4.5 | 0.6 | 0.3 |
| Fucosylation | A-fucosylated | 5.2 | 4.8 | 4.5 | 0.6 | 0.3 |
|  | Mono-fucosylated | 92.0 | 92.5 | 92.3 | 97.3 | 97.4 |
|  | Bi-fucosylated | 2.8 | 2.7 | 3.3 | 2.2 | 2.3 |
| Sialylation | Mono-sialylated | 26.1 | 26.8 | 25.4 | 21.6 | 21.6 |
|  | Di-sialylated | 55.7 | 56.2 | 57.1 | 54.9 | 55.6 |
|  | Tri-sialylated | 10.2 | 10.0 | 10.8 | 19.2 | 18.4 |
|  | Tetra-sialylated | 0.4 | 0.3 | 0.0 | 1.7 | 1.9 |
