# Supplementary material for: In-vivo biological activity and glycosylation analysis of a biosimilar recombinant human follicle-stimulating hormone product (Bemfola) compared with its reference medicinal product (GONAL-f)
Source: PLoS One. 2017 Sep 7;12(9):e0184139. doi: 10.1371/journal.pone.0184139 (PMC5589168; doi:10.1371/journal.pone.0184139)
Supplement: S6 Table — (DOCX) [file pone.0184139.s007.docx]

**S6 Table. Bemfola bioactivity (*in vivo*)**

| **Batch number** | **Nominal specific activity** | **% of nominal value** | **Specific activity IU/nominal mg** |
| --- | --- | --- | --- |
| **PNS30388** | 13636 | 111 | 15272 |
| **PNS30230** | 13636 | 107 | 14727 |
| **PPS30400** | 13636 | 116 | 15954 |
| **PPS30021** | 13636 | 104 | 13909 |
| **PNS30390** | 13636 | 96 | 13227 |
| **PNS30228** | 13636 | 105 | 14318 |
| **PNS30389** | 13636 | 91 | 12409 |
| **PNS30229B** | 13636 | 111 | 15409 |
| **Average (CV%)** |  | 105.6 (8.3) | 14403 (8.3) |
