# Supplementary material for: In-vivo biological activity and glycosylation analysis of a biosimilar recombinant human follicle-stimulating hormone product (Bemfola) compared with its reference medicinal product (GONAL-f)
Source: PLoS One. 2017 Sep 7;12(9):e0184139. doi: 10.1371/journal.pone.0184139 (PMC5589168; doi:10.1371/journal.pone.0184139)
Supplement: S7 Table — (DOCX) [file pone.0184139.s008.docx]

**S7 Table. GONAL-f bioactivity (*in vivo*)**

| **Batch number** | **Nominal specific activity** | **% of nominal value** | **Specific activity IU/nominal mg** |
| --- | --- | --- | --- |
| **1** | **13636** | 90 | 12272 |
| **2** | **13636** | 89 | 12136 |
| **3** | **13636** | 96 | 13091 |
| **4** | **13636** | 97 | 13227 |
| **5** | **13636** | 96 | 13091 |
| **6** | **13636** | 98 | 13363 |
| **7** | **13636** | 90 | 12272 |
| **8** | **13636** | 100 | 13636 |
| **9** | **13636** | 99 | 13500 |
| **10** | **13636** | 90 | 12272 |
| **11** | **13636** | 101 | 13772 |
| **12** | **13636** | 102 | 13909 |
| **13** | **13636** | 102 | 13909 |
| **14** | **13636** | 94 | 12818 |
| **15** | **13636** | 105 | 14318 |
| **16** | **13636** | 102 | 13909 |
| **17** | **13636** | 110 | 15000 |
| **18** | **13636** | 93 | 12681 |
| **19** | **13636** | 94 | 12818 |
| **20** | **13636** | 92 | 12545 |
| **21** | **13636** | 105 | 14318 |
| **22** | **13636** | 96 | 13091 |
| **Average (CV%)** |  | 97.3 (5.8) | 13270 (5.8) |
