# Supplementary material for: In-vivo biological activity and glycosylation analysis of a biosimilar recombinant human follicle-stimulating hormone product (Bemfola) compared with its reference medicinal product (GONAL-f)
Source: PLoS One. 2017 Sep 7;12(9):e0184139. doi: 10.1371/journal.pone.0184139 (PMC5589168; doi:10.1371/journal.pone.0184139)
Supplement: S8 Table — (DOCX) [file pone.0184139.s009.docx]

# S8 Table. Summary statistics for relative % nominal value

| Site | Count | Average | Median | Standard deviation | CV% |
| --- | --- | --- | --- | --- | --- |
| Bemfola | 8 | 105.625 | 106.5 | 8.71677 | 8.25257 |
| GONAL-f | 22 | 97.3182 | 96.5 | 5.66011 | 5.81608 |
| Total | 30 | 99.5333 | 98.5 | 7.44976 | 7.48468 |
