# Supplementary material for: In-vivo biological activity and glycosylation analysis of a biosimilar recombinant human follicle-stimulating hormone product (Bemfola) compared with its reference medicinal product (GONAL-f)
Source: PLoS One. 2017 Sep 7;12(9):e0184139. doi: 10.1371/journal.pone.0184139 (PMC5589168; doi:10.1371/journal.pone.0184139)
Supplement: S9 Table — (DOCX) [file pone.0184139.s010.docx]

S9 Table. ANOVA Table for Final Result (Relative %) by Site

| **Source** | **Sum of Squares** | **Df** | **Mean Square** | **F-Ratio** | **P-Value** |
| --- | --- | --- | --- | --- | --- |
| Between groups | 404.819 | 1 | 404.819 | 9.41 | 0.0048 |
| Within groups | 1204.65 | 28 | 43.0231 |  |  |
| Total (Corr.) | 1609.47 | 29 |  |  |  |

The ANOVA table splits the variance of the final result (relative %) into two components: a between-group component and a within-group component. The F-ratio, which in this case equals 9.40933, is the ratio of the between-group estimate to the within-group estimate. Since the P-value of the F-test is less than 0.05, there is a statistically significant difference between the mean final result (relative %) from one level of each preparation to another, at the 95.0% confidence level.
