# Supplementary material for: In-vivo biological activity and glycosylation analysis of a biosimilar recombinant human follicle-stimulating hormone product (Bemfola) compared with its reference medicinal product (GONAL-f)
Source: PLoS One. 2017 Sep 7;12(9):e0184139. doi: 10.1371/journal.pone.0184139 (PMC5589168; doi:10.1371/journal.pone.0184139)
Supplement: S10 Table — (DOCX) [file pone.0184139.s011.docx]

**S10 Table. Variance Check**

|  | **Test** | **P-Value** |
| --- | --- | --- |
| Bartlett's | 1.08215 | 0.14709 |

The statistic displayed in this table tests the null hypothesis that the standard deviations of the final result (relative %) within each of the two preparations is the same. Of particular interest is the P-value. Since the the P-value is greater than or equal to 0.05, there is not a statistically significant difference amongst the standard deviations at the 95.0% confidence level.
