# Supplementary material for: In-vivo biological activity and glycosylation analysis of a biosimilar recombinant human follicle-stimulating hormone product (Bemfola) compared with its reference medicinal product (GONAL-f)
Source: PLoS One. 2017 Sep 7;12(9):e0184139. doi: 10.1371/journal.pone.0184139 (PMC5589168; doi:10.1371/journal.pone.0184139)
Supplement: S11 Table — (DOCX) [file pone.0184139.s012.docx]

**S11 Table. Multiple Range Tests for Cross-Validation (Relative %) and Variance Check per Subgroup**

Multiple range tests

| *Column 3* | *Count* | *Mean* | *Homogeneous groups* |
| --- | --- | --- | --- |
| GONAL-f Group C | 8 | 96.125 | X |
| GONAL-f Group A | 7 | 97.5714 | X |
| GONAL-f Group B | 7 | 98.4286 | X |
| Bemfola | 8 | 105.625 | X |

| *Contrast* | *Sig* | *Difference* | *+/− Limits* |
| --- | --- | --- | --- |
| GONAL-f Group A – GONAL-f Group B |  | –0.857143 | 7.41502 |
| GONAL-f Group A − Bemfola | * | –8.05357 | 7.17957 |
| GONAL-f Group A – GONAL-f Group C |  | 1.44643 | 7.17957 |
| GONAL-f Group B − Bemfola | * | –7.19643 | 7.17957 |
| GONAL-f Group B – GONAL-f Group C |  | 2.30357 | 7.17957 |
| Bemfola – GONAL-f Group C | * | 9.5 | 6.93612 |

*Denotes a statistically significant difference

The analysis in this table applies a multiple comparison procedure to determine which means are significantly different from which others. In the top half of the output, two homogeneous groups are identified using columns of Xs. Within each column, the levels containing X form a group of means within which there are no statistically significant differences. The method currently being used to discriminate among the means is Fisher’s least significant difference procedure. With this method, there is a 5.0% risk of calling each pair of means significantly different when the actual difference is zero. The bottom half of the output shows the estimated difference between each pairs of means. An asterisk has been placed next to three pairs, indicating that these pairs show statistically significant differences at the 95% confidence interval level.

Variance check

|  | *Test* | *P-value* |
| --- | --- | --- |
| Bartlett’s | 1.19319 | 0.229476 |

The analysis shown in this table tests the null hypothesis that the standard deviations of relative % within each of the four levels of Column 3 is the same. Of particular interest is the P-value. Since the P-value is greater than 0.05, there is not a statistically significant difference among the standard deviations at the 95% confidence level interval.
