# Supplementary material for: In-vivo biological activity and glycosylation analysis of a biosimilar recombinant human follicle-stimulating hormone product (Bemfola) compared with its reference medicinal product (GONAL-f)
Source: PLoS One. 2017 Sep 7;12(9):e0184139. doi: 10.1371/journal.pone.0184139 (PMC5589168; doi:10.1371/journal.pone.0184139)
Supplement: S12 Table — (DOCX) [file pone.0184139.s013.docx]

# S12 Table. Multiple Range Tests for Final Result (Relative %) by Site

| **Col_3** | **Count** | **Mean** | **Homogeneous Groups** |
| --- | --- | --- | --- |
| 2 | 22 | 97.3182 | X |
| 1 | 8 | 105.625 | X |

| **Contrast** | **Sig.** | **Difference** | **+/- Limits** |
| --- | --- | --- | --- |
| 1 - 2 | * | 8.30682 | 5.54718 |

*Denotes a statistically significant difference. Method: 95.0% least significant difference.

The analysis in this table applies a multiple comparison procedure to determine which means are significantly different from which others. The bottom half of the output shows the estimated difference between each pair of means. The asterisk indicates that this pair shows a statistically significant difference at the 95.0% confidence level. In the top of the output, two homogenous groups are identified (denoted by X). Within each column, the levels form a group of means within which there are no statistically significant differences. The method currently being used to discriminate among the means is Fisher's least significant difference procedure. With this method, there is a 5.0% risk of calling each pair of means significantly different when the actual difference equals 0.
