# Supplementary material for: In-vivo biological activity and glycosylation analysis of a biosimilar recombinant human follicle-stimulating hormone product (Bemfola) compared with its reference medicinal product (GONAL-f)
Source: PLoS One. 2017 Sep 7;12(9):e0184139. doi: 10.1371/journal.pone.0184139 (PMC5589168; doi:10.1371/journal.pone.0184139)
Supplement: S5 Fig — (DOCX) [file pone.0184139.s018.docx]

# S5 Figure. Antennarity distribution (%) by LC-MS analysis in GONAL-f and Bemfola batches on Asn24
