# Supplementary material for: In-vivo biological activity and glycosylation analysis of a biosimilar recombinant human follicle-stimulating hormone product (Bemfola) compared with its reference medicinal product (GONAL-f)
Source: PLoS One. 2017 Sep 7;12(9):e0184139. doi: 10.1371/journal.pone.0184139 (PMC5589168; doi:10.1371/journal.pone.0184139)
Supplement: S7 Fig — (DOCX) [file pone.0184139.s020.docx]

**S7 Figure. Sample-Size Determination**

#
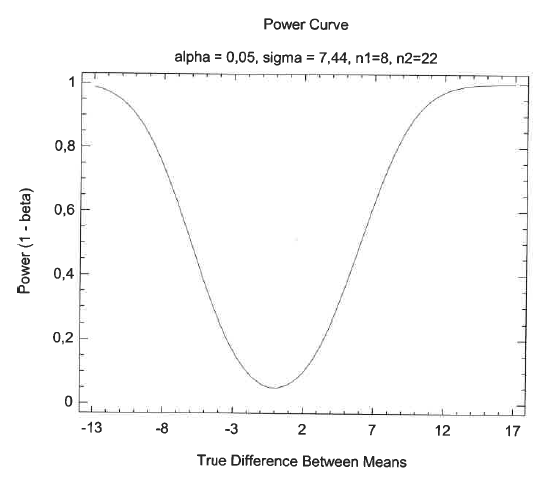


Parameter to be estimated: difference between two normal means

Desired power: 75.0% for difference=0.0 versus difference=8.3

Type of alternative: not equal

Alpha risk: 5.0%

Sigma: 7.44 (known)

The required sample size is 8 observations from sample 1 and 22 observations from sample 2.

This procedure determines the sample size required when comparing the means of two normal distributions. Assuming that the standard deviation of the normal distribution is equal to 7.44, 8 observations are required in sample 1and 22 in sample 2 to have a 75.0% chance of rejecting the hypothesis that mul-mu2=0.0 when the true mul-mu2=8.3 (using a two-sided test).
